# Nanoengineered Photoactive Micromotors for Targeted Pollutant Capture, Degradation, and SERS-Based Detection

**DOI:** 10.34133/research.1110

**Published:** 2026-02-09

**Authors:** Viktoria D. Lovasz, João M. Gonçalves, Gail A. Vinnacombe-Willson, Luis M. Liz-Marzán, Katherine Villa

**Affiliations:** ^1^ Institute of Chemical Research of Catalonia (ICIQ-CERCA), The Barcelona Institute of Science and Technology (BIST), Tarragona, Spain.; ^2^ Universitat Rovira i Virgili (URV), Campus Sescelades, Tarragona, Spain.; ^3^ CIC biomaGUNE, Basque Research and Technology Alliance (BRTA), Donostia-San Sebastián, Spain.; ^4^ CIBER de Bioingeniería, Biomateriales y Nanomedicina (CIBER-BBN), Donostia-San Sebastián, Spain.; ^5^ Ikerbasque, Basque Foundation for Science, Bilbao, Spain.; ^6^ Institució Catalana de Recerca i Estudis Avançats (ICREA), Barcelona, Spain.

## Abstract

Achieving both selective pollutant degradation and real-time detection within a single micromotor system remains challenging for environmental monitoring. To address this limitation, we engineered gold-nanostar-decorated, molecularly imprinted BiVO_4_ micromotors that combine simultaneous capture, photocatalytic degradation, and in situ detection of pollutants via surface-enhanced Raman spectroscopy (SERS). Plasmonic gold nanostars provide strong SERS enhancement for real-time tracking of pollutant degradation, while micromotors maintain autonomous propulsion under visible light irradiation. Surface molecular imprinting ensures selective recognition of rhodamine 6G and synergistically improves both photocatalytic and sensing performance. This multifunctional design establishes an all-in-one micromotor platform that bridges environmental remediation and on-board monitoring, opening opportunities for advanced water treatment technologies.

## Introduction

The integration of nanotechnology with environmental and analytical sciences has led to marked innovations in the design and applications of micro- and nanomotors (MNMs) [1–3]. These tiny micromachines, which move in liquid environments using chemical fuels or external fields, hold great promise for applications ranging from environmental remediation and sensing to medical diagnostics [1,4,5]. In particular, light-driven micro/nanomotors (LMNMs), based on photocatalytic materials, stand out because of their ability to harness light as a clean, abundant, and renewable energy source for propulsion [6–8]. Such photoactive systems have been broadly explored for environmental remediation and solar-driven technologies [4,9,10], where their autonomous self-propulsion enhances mass transfer and accelerates pollutant degradation.

Unlike Janus micromotor design, LMNMs based on single-component semiconductors with anisotropic shapes offer a simple yet effective approach because their intrinsic asymmetry enables self-propulsion while simultaneously providing photocatalytic activity [1]. Among these materials, bismuth vanadate (BiVO_4_) has received particular attention owing to its visible-light absorption arising from a favorable bandgap (around 2.4 eV) and its straightforward fabrication into diverse anisotropic morphologies, including rough spheres, butterfly-like, and star-shaped structures [11–19]. In particular, star-shaped BiVO_4_ exhibits enhanced photocatalytic performance compared to other morphologies, due to the increased exposure of highly reactive {010} facets [18]. Furthermore, the star-shaped morphology intrinsically breaks symmetry, often featuring an elongated tip and a rough surface, which promote asymmetric reaction gradients under illumination. These properties render star-shaped BiVO_4_ an excellent model system for developing visible-light-driven photocatalytic micromotors.

Recent efforts to optimize micromotor-based remediation have focused on improving both selectivity and photocatalytic performance. Surface molecular imprinting polymers (MIPs) represent a particularly effective strategy: Introducing molecularly defined recognition sites on the micromotor surface enables selective pollutant binding and may facilitate more efficient photocatalytic degradation [11,20–22]. However, these systems still rely on separate analytical methods to verify pollutant degradation. Realizing photoactive micromotors that can simultaneously degrade and sense pollutants in real time remains a key step toward autonomous environmental monitoring.

Conventional colorimetric and ultraviolet–visible (UV–Vis) spectrophotometric methods are low cost and simple but generally lack molecular specificity and sensitivity, while fluorescence- and chemiluminescence-based techniques often require labeling and more complex sample preparation [23–26]. In contrast, surface-enhanced Raman spectroscopy (SERS) enables ultrasensitive detection of molecular vibrational fingerprints and real-time monitoring of chemical transformations [27,28]. SERS relies on localized surface plasmon resonances in plasmonic nanostructures [29,30], with gold nanostars (AuNSt) being particularly effective because of their branched morphology and sharp tips that generate abundant electromagnetic “hotspots”, which enable ultrasensitive detection, in some cases down to the single-molecule level. This capability is particularly relevant for environmental monitoring of priority pollutants such as microplastics [31–33]. Therefore, integrating such plasmonic structures into micromotors offers a direct pathway to combine active transport, pollutant degradation, and simultaneous sensitive sensing [34–42].

Previous studies have explored micromotors as SERS-active probes using diverse material configurations, including silver nanowire@SiO_2_ [38] Fe_2_O_3_@Ag nanoparticles [40], MXene-Fe_2_O_3_ magnetic micromotors [39], urease-powered Au-coated enzymatic micromotors [41], and Au-decorated MoS_2_ light-driven micromotors [42]. Although these systems demonstrate effective signal enhancement, they often suffer from limitations such as material instability, reliance on UV-driven propulsion or complex magnetic control, and a lack of photocatalytic degradation capability. Moreover, these works focus primarily on SERS sensing without addressing pollutant decomposition. Consequently, the design of a visible-light-driven micromotor that integrates pollutant capture, photocatalytic degradation, and in situ SERS monitoring within a single self-propelled system remains challenging.

**Fig. 1.**
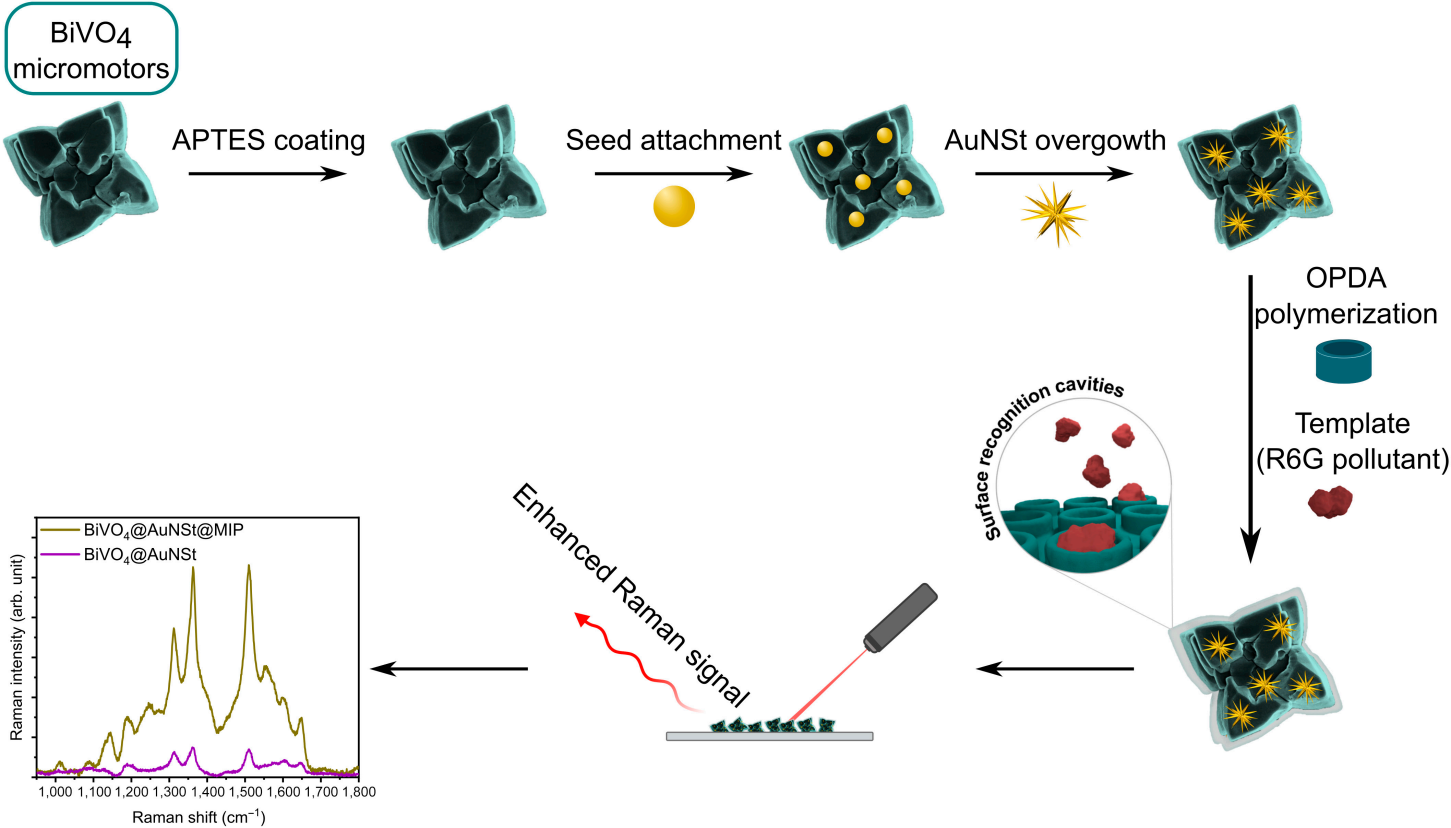
Schematic representation of the stepwise synthesis of AuNSt-functionalized BiVO_4_ micromotors, including colloidal gold seed attachment, in situ overgrowth of the attached seeds into AuNSt, and final surface modification with OPDA to form the molecularly imprinted layer. R6G was used as the template molecule. The resulting BiVO_4_@AuNSt@MIP micromotors were subsequently used for the selective SERS detection of R6G. arb. unit., arbitrary units.

Here, we present a multifunctional micromotor that integrates autonomous propulsion, pollutant degradation, and in situ sensing within a single platform. Specifically, star-shaped BiVO_4_ micromotors were functionalized with AuNSt and a molecularly imprinted polymer (BiVO_4_@AuNSt@MIP) for the capture, photocatalytic degradation, and in situ SERS detection of rhodamine 6G (R6G) as a model pollutant. In this system, BiVO_4_ provides visible-light-driven propulsion and photocatalytic activity in the presence of H_2_O_2_, while AuNSt enable ultrasensitive SERS detection, and the imprinted layer confers molecular selectivity toward the target pollutant (Fig. 1). This work demonstrates a strategy to synergistically combine pollutant capture, degradation, and simultaneous monitoring within a single self-propelled micromotor, with implications for environmental remediation and sensing, while creating new avenues for the rational design of multifunctional photoactive micromotors.

## Results and Discussion

Star-shaped BiVO_4_ micromotors were prepared as previously reported with minor modifications [14] and subsequently functionalized with AuNSt using an in situ seeded growth strategy. In this approach, small gold seeds were first synthesized as a colloidal dispersion and then anchored onto the BiVO_4_ surface via a linker molecule, followed by wet chemical overgrowth into branched nanostructures, directly on the micromotor surface. Although stepwise seed-mediated growth is well established in colloidal synthesis to tailor nanoparticle morphology [43] and has been previously adapted for oxide substrates [44,45], this work demonstrates the application of such a strategy on self-propelled micromotors. This strategy enables the controlled formation of plasmonic nanostructures directly on photocatalytic BiVO_4_ micromotors (Fig. 1). Briefly, small gold nanoparticle seeds coated with cetyltrimethylammonium chloride (CTAC) were first prepared in colloidal dispersion following a previously reported method [46]. Meanwhile, BiVO_4_ micromotors were functionalized with an aminopropyl-trimethoxy silane (APTES) layer containing primary amine groups that serve as anchoring points for the colloidal Au seeds [47,48]. The seed solution was thus incubated for 15 min with the APTES-functionalized BiVO_4_ microparticles under sonication to facilitate seed attachment. In the last step, BiVO_4_-bound gold seeds were overgrown into AuNSt by transferring them into a growth solution containing shape directing agents Triton X-100, AgNO_3_, HCl, additional gold precursor (HAuCl_4_), and ascorbic acid as a weak reductant. Our synthetic approach enabled us to tune the AuNSt coverage density by altering the seed solution and growth solution concentrations, resulting in 2 types of AuNSt-coated micromotors: “low-coverage” and “high-coverage” samples (Fig. S1). Detailed synthesis procedures are provided in Materials and Methods.

**Fig. 2.**
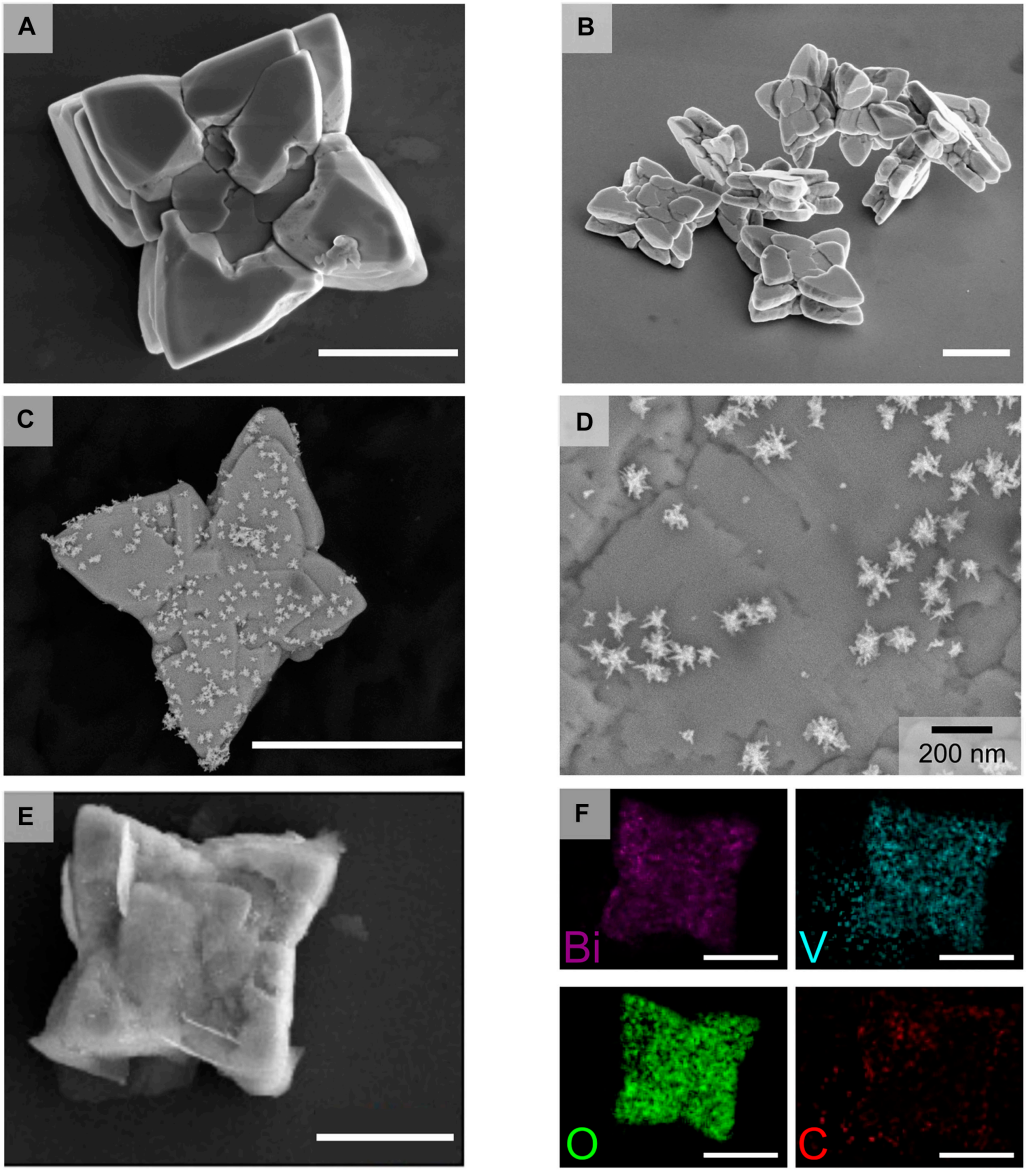
Morphological and compositional characterization of star-shaped BiVO_4_ micromotors. (A and B) FESEM images of pristine BiVO_4_ micromotors. (C and D) BiVO_4_@AuNSt micromotors showing the formation of branched Au nanostars on the surface. (E) BiVO_4_@AuNSt@MIP after polymerization. (F) EDS elemental mapping of BiVO_4_@AuNSt@MIP. Scale bars, 2 μm (unless labeled otherwise).

The morphology of the synthesized BiVO_4_ micromotors was characterized by field-emission scanning electron microscopy (FESEM). As shown in Fig. 2A and B, the micromotors exhibited a star-shaped morphology and sizes ranging from 3 to 5 μm (average microparticle size, 4.2 ± 0.2 μm; see Fig. S2A). As shown in Fig. 2C and D, SEM imaging confirmed the direct formation of highly branched AuNSt on the surface of the micromotors after synthesis. The average AuNSt tip-to-tip length was 100 ± 30 nm for low-coverage samples and 140 ± 30 nm for high-coverage samples, with corresponding size distributions provided in Fig. S2B and C. Energy-dispersive x-ray spectroscopy (EDS) mapping (Fig. S3) revealed distinct Au signals distributed over the micromotor surface, confirming the in situ formation of AuNSt. In addition, the AuNSt surface coverage was quantified from SEM image analysis, yielding an average density of 57.1 ± 15.6 AuNSt/μm^2^ for the high-coverage samples and 38.0 ± 4.0 AuNSt/μm^2^ for the low-coverage samples.

Subsequently, BiVO_4_@AuNSt@MIP micromotors were obtained by surface functionalization with *o*-phenylenediamine (OPDA) as the functional monomer and R6G as the template molecule for MIP formation [11,49]. A light-induced polymerization process was used to form a thin MIP layer, followed by template removal to generate NH_2_-enriched surface recognition sites that would enhance the degradation efficiency of the micromotors toward R6G. After MIP functionalization, an additional thin coating was observed on the micromotor surface (Fig. 2E and Fig. S4). EDS analysis confirmed the presence of C associated with this layer (Fig. 2F), supporting OPDA polymerization and successful surface functionalization [11]. EDS signals for Bi, V, and O were found to be homogeneously distributed throughout the micromotor, which is consistent with the BiVO_4_ scaffold. Although Au was not clearly distinguishable in the elemental map after MIP coating (Fig. 2F), point EDS spectra verified its continued presence, indicating that the AuNSt remained on the micromotor surface after polymer deposition (Fig. S5).

**Fig. 3.**
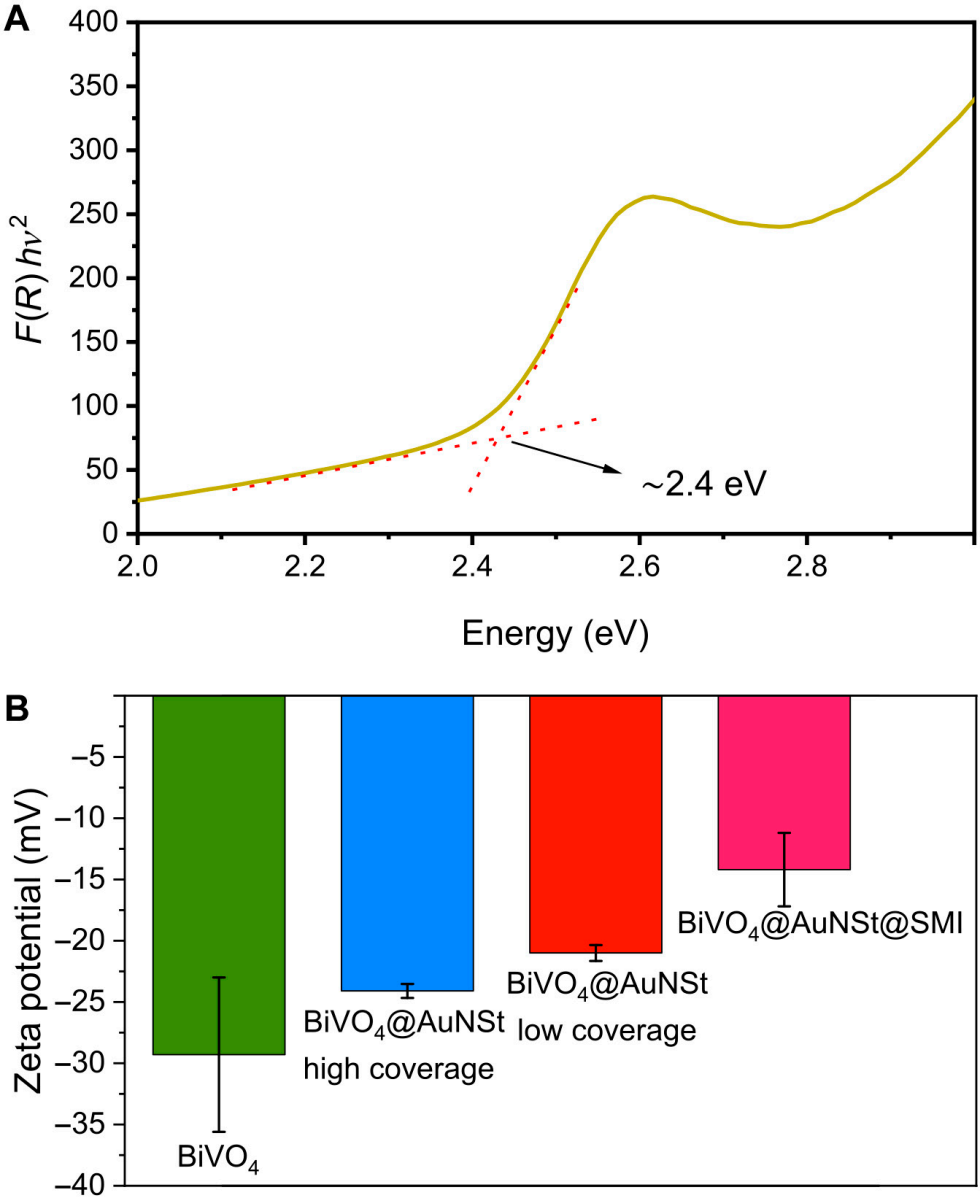
Optical and surface characterization of star-shaped BiVO_4_ micromotors. (A) Tauc plot from which the bandgap of BiVO_4_ micromotors can be determined. (B) Zeta potential values for BiVO_4_, BiVO_4_@AuNSt (high and low surface coverage), and BiVO_4_@AuNSt@MIP micromotors. Error bars represent the standard deviation calculated for *n* = 3.

The bandgap of star-shaped BiVO_4_ micromotors was determined by UV–Vis diffuse reflectance spectroscopy using an integrating sphere. After processing the data with the Kubelka–Munk function, a Tauc plot served to estimate a bandgap at approximately 2.4 eV, consistent with previous reports (Fig. 3A) [13,50]. X-ray diffraction (XRD) analysis (Fig. S6) revealed diffraction peaks corresponding to the monoclinic phase (powder diffraction file: 01-075-1867), which is known to display superior photoactivity compared to the tetragonal phase [51,52]. Moreover, major changes in zeta potential were observed after sequential functionalization with AuNSt and MIP (Fig. 3B). The bare BiVO_4_ micromotors exhibited a zeta potential of −29.3 mV, consistent with surface hydroxyl groups [49]. Upon coating with AuNSt, the apparent surface charge, as reflected by the zeta potential, became less negative because of the introduction of positively charged protonated amino groups from APTES modification. The zeta potential increased to −24.1 mV for high AuNSt coverage, whereas low coverage resulted in a slightly less negative value (−21.0 mV). This difference suggests that lower coverage exposes a greater fraction of the underlying APTES layer, allowing more amino groups to influence the zeta potential. Following MIP functionalization, the potential shifted to −11.3 mV, indicating the incorporation of additional amino groups from the OPDA-based polymer and confirming the successful surface modification aimed at generating R6G recognition sites.

To further verify the presence of MIP, we performed the thermogravimetric analysis (TGA), as shown in Fig. S7. It revealed an approximate 0.6% weight loss between 275 and 500 °C, which is attributed to the decomposition of the polymeric MIP layer. BiVO_4_ is known to be thermally stable within this temperature range, confirming that the observed mass loss originates from the surface-bound organic coating [53–55]. The low polymer content is consistent with a thin MIP layer that preserves micromotor propulsion and photocatalytic activity.

Anisotropic BiVO_4_ micromotors are known to exhibit autonomous motion under visible light irradiation, particularly for star-shaped morphologies such as those obtained in this study [13,14,16,56]. Next, we qualitatively evaluated the micromotor motion capabilities under different experimental conditions. In pure water, the micromotors displayed random Brownian motion, regardless of light exposure. In contrast, in the presence of both light and H_2_O_2_ as a chemical fuel, the micromotors displayed active propulsion, as evidenced by the extended trajectories shown in Movie S1. In addition, by varying the fuel concentration, we determined that a minimum of 0.1% H_2_O_2_ was required to achieve consistent autonomous motion (Movie S2). Importantly, no bubbles were observed under any of the conditions described, thus excluding self-propulsion due to bubble formation.

The propulsion mechanism of BiVO_4_-based micromotors has been widely discussed in the literature [14,16,57]. Upon visible light illumination, BiVO_4_ generates electron-hole (e^−^/h^+^) pairs that separate across different crystalline facets, leading to spatially heterogeneous surface redox activity. In the presence of H_2_O_2_, these photogenerated charge carriers participate in photocatalytic reactions that produce H_2_O and O_2_ at the micromotor surface [Eqs. (1) and (2)].

$$H_{2}O_{2}+2h^{+}\to O_{2}+2H^{+}$$ (1)

$$H_{2}O_{2}+2H^{+}+2e^{-}\to 2H_{2}O$$ (2)

On the basis of previous studies on anisotropic BiVO_4_ micromotors, motion is commonly attributed to a phoretic mechanism driven by asymmetric photocatalytic reactions at the particle surface, rather than to gas evolution. Although direct measurements of local ionic or pH gradients were not performed here, the observed light-dependent propulsion behavior, fuel concentration, particle asymmetry, and AuNSt coverage is fully consistent with a self-electrophoretic propulsion mechanism reported for similar BiVO_4_-based systems. In addition to these reactions, secondary pathways may generate reactive oxygen species (ROS), such as hydroxyl and superoxide radicals, which are relevant for photocatalytic degradation.

**Fig. 4.**
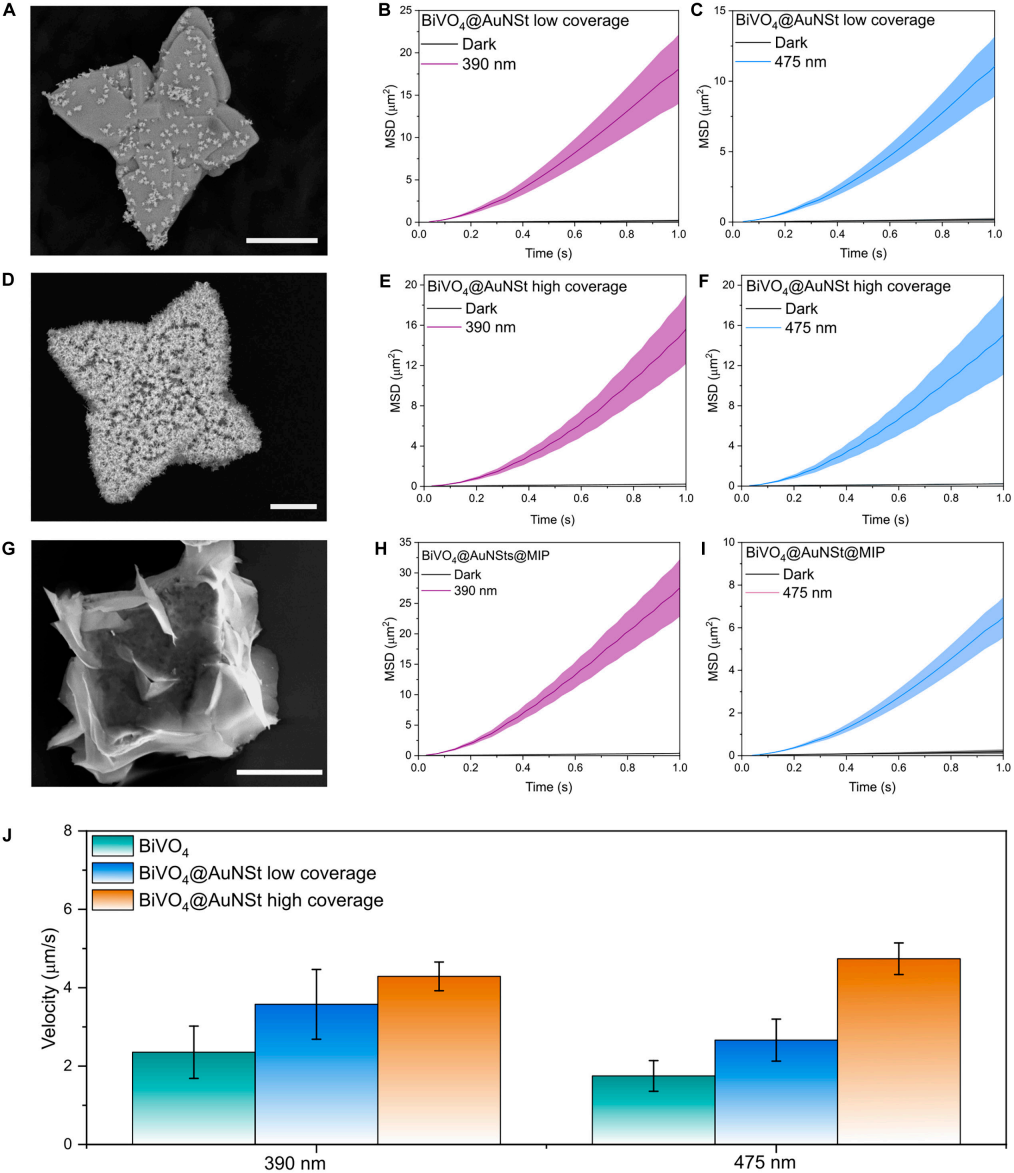
Motion characterization of BiVO_4_-based micromotors. (A) SEM image of low-density BiVO_4_@AuNSt micromotors. (B) MSD analysis of low-density BiVO_4_@AuNSt micromotors under UV excitation. (C) MSD analysis of low-density BiVO_4_@AuNSt micromotors under blue light excitation. (D) SEM image of high-density BiVO_4_@AuNSt micromotors. (E) MSD analysis of high-density BiVO_4_@AuNSt micromotors under UV excitation. (F) MSD analysis of high-density BiVO_4_@AuNSt micromotors under blue light excitation. (G) SEM image of BiVO_4_@AuNSt@MIP micromotors. (H) MSD analysis of BiVO_4_@AuNSt@MIP micromotors under UV excitation. (I) MSD analysis of BiVO_4_@AuNSt@MIP micromotors under blue light excitation. (J) Comparison of micromotor velocity according to AuNSt coating density. Shadowed regions in the MSD plots and error bars represent the standard deviation from the mean for *n* > 20. Scale bars, 1 μm.

To quantify the propulsion performance, we evaluated the active motion through the mean square displacement (MSD), where a linear behavior in the short-time regime would correspond to purely Brownian motion, whereas a parabolic profile would be indicative of self-propulsion [58–61]. Figure 4B and C, as well as E and F, shows the MSD profiles of BiVO_4_@AuNSt micromotors with different coverage densities under 390- and 475-nm excitation. For both wavelengths, the recorded parabolic MSD profiles confirmed active propulsion, indicating that the presence of AuNSt do not hinder light-driven motion. Similarly, MIP surface functionalization did not hamper either the self-propulsion behavior, as shown in Fig. 4G to I and Movie S3.

We observed that the propulsion velocity of the micromotors was affected by AuNSt coverage density (Fig. 4J and Table S1), with higher AuNSt loadings leading to faster motion under visible light irradiation. This effect can be attributed to the role of Au acting as an efficient electron sink, which decreases charge pair recombination in BiVO_4_ [62,63]. This enhanced charge separation strengthens the asymmetric chemical gradients responsible for light-driven propulsion, resulting in higher velocities for micromotors with increased AuNSt coverage, in agreement with previous reports on metal–semiconductor micromotors [64–68].

Beyond individual motion, the micromotors exhibit reversible collective behavior, transitioning between dispersion and clustering under light stimulus (Movie S4 and Fig. S8). This collective behavior is consistent with phoretic interactions mediated by photocatalytically generated chemical fields. When illuminated, BiVO_4_ micromotors generate local solute gradients in the surrounding fluid, which induce effective attractive interactions between neighboring particles and promote dynamic clustering [63].

**Fig. 5.**
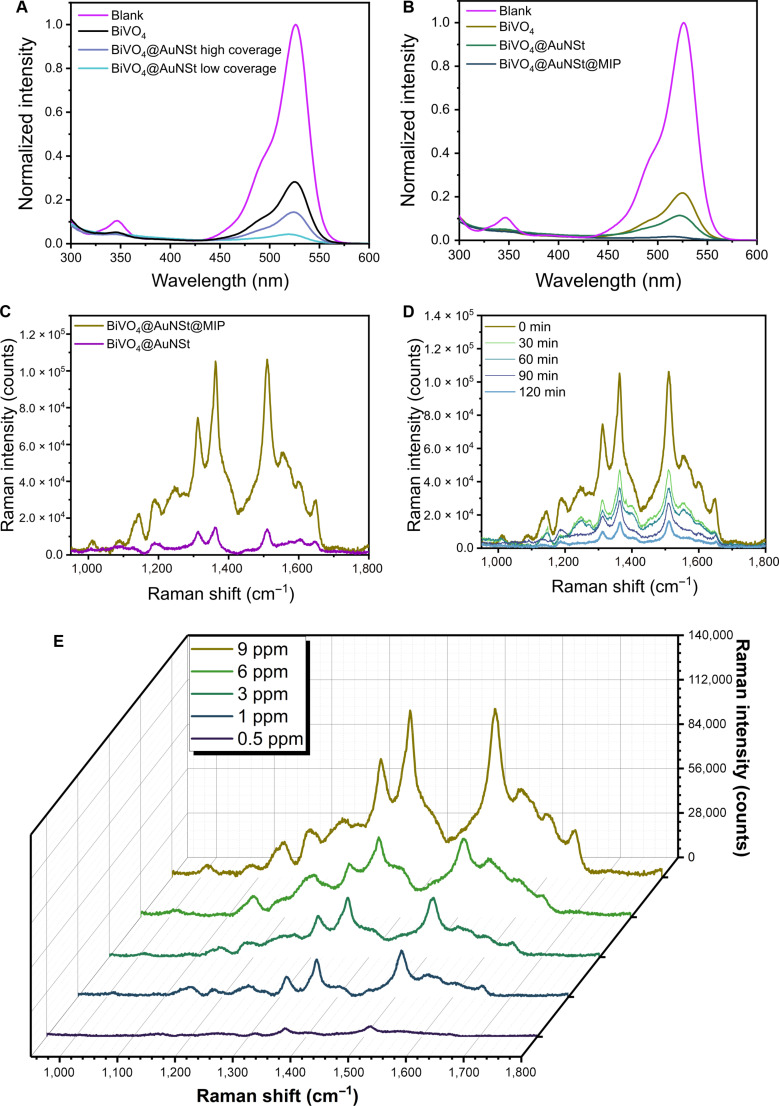
Photocatalytic degradation and SERS detection of R6G by BiVO_4_-based micromotors. (A) UV–Vis spectra comparing different AuNSt coating densities after 90 min of photocatalytic degradation. (B) UV–Vis spectra showing the degradation profile by micromotors at each stage of material modification. The blank measurements correspond to a 5-ppm R6G solution without micromotors. (C) SERS spectra of BiVO_4_@AuNSt and BiVO_4_@AuNSt@MIP in a 9-ppm R6G solution. (D) Time-dependent SERS spectra showing photocatalytic degradation of R6G overtime. (E) SERS spectra of R6G at varying concentrations in the presence of BiVO_4_@AuNSt@MIP micromotors.

After characterizing the autonomous motion of the photocatalytic micromotors, we next evaluated the R6G degradation performance of AuNSt-coated micromotors, comparing low and high coverage (Fig. 5A). Low-coverage AuNSt-decorated micromotors showed enhanced degradation compared to bare BiVO_4_ under visible light irradiation (90 min), which can be attributed to the synergistic effects of higher motion velocities and Au-catalyzed H_2_O_2_ decomposition generating ROS, including ·OH and ·O_2_^−^, which substantially enhance oxidative degradation of organic pollutants [63]. To gain further insight into the degradation mechanism, we investigated the role of radical quenchers on the photocatalytic performance and found that superoxide radicals were the dominant contributors to R6G degradation (Fig. S9).

On the other hand, micromotors with high AuNSt coverage resulted in reduced photocatalytic efficiency, likely due to partial blocking of the active sites of BiVO_4_ micromotors. Consequently, micromotors with low AuNSt coverage were selected for subsequent SERS experiments because they provided the best balance between photocatalytic performance and light-driven propulsion for the 2 types of AuNSt-decorated micromotors tested.

To quantify gold loading and its associated cost, we used inductively coupled plasma mass spectrometry (ICP-MS). The analysis revealed trace gold loadings in the range of 0.04 to 0.2 ng, corresponding to an estimated gold cost of ~0.42 euros per gram of BiVO_4_ microparticles under the optimized low-coverage conditions. For comparison, a 10-fold higher AuNSt coverage would increase the gold cost to ~16.7 euros per gram of BiVO_4_, without providing additional photocatalytic benefit.

Interestingly, BiVO_4_@AuNSt@MIP micromotors achieved an even higher degradation efficiency within 120 min of irradiation (Fig. 5B). This improvement is attributed to the presence of molecular recognition sites introduced by the imprinting process, which enhance the affinity of the micromotors toward R6G molecules, indicating that the incorporation of AuNSt and the MIP layer presents clear advantages in pollutant degradation.

A detailed comparison between this system and previously reported micromotors used for R6G degradation is provided in Table S2. Most existing approaches rely on UV light excitation [69–71] and/or require relatively high H_2_O_2_ concentrations and surfactants such as sodium dodecyl sulfate (SDS) to promote bubble-driven propulsion [69,70,72–75]. In contrast, the BiVO_4_@AuNSt@MIP micromotors move under visible light irradiation at low fuel concentration and without any additives, while simultaneously enabling sensitive in situ detection coupled with photocatalytic degradation.

More importantly, the photoactive micromotors exhibited strong SERS activity due to the presence of plasmonic AuNSt. Distinct Raman peaks were observed at 1,358 and 1,510 cm^−1^, corresponding to vibrational modes of the aromatic rings in R6G (Fig. 5C) [76]. When the micromotors were further modified with the MIP layer, an even stronger signal was recorded, attributed to the presence of specific recognition sites that accumulate R6G molecules in close proximity to the AuNSt surface (Fig. 5C). Although one could expect the polymeric layer to attenuate SERS intensity by increasing the analyte–plasmon distance, our results revealed the opposite effect, indicating that molecular imprinting facilitates local enrichment of R6G at the plasmonic hotspots [77]. Specifically, at the characteristic 1,358 cm^−1^ peak of R6G, BiVO_4_@AuNSt@MIP micromotors exhibited an 87% higher signal compared to BiVO_4_@AuNSt micromotors without the MIP layer, demonstrating the synergistic interaction between molecular recognition and plasmonic enhancement. In contrast, in the absence of AuNSt, no Raman signal was detected for BiVO_4_ micromotors in a R6G solution of 5 parts per million (ppm) (Fig. S10).

The degradation of R6G was further monitored using SERS, providing insights into the degradation profile and confirming the enhanced affinity and catalytic performance of the MIP-coated micromotors. SERS-based monitoring of the photocatalytic degradation of R6G at regular time intervals is shown in Fig. 5D. At time zero, the spectrum exhibits multiple sharp and intense Raman bands characteristic of R6G. After 120 min of visible light exposure, the SERS intensity decreases markedly across all characteristic peaks, indicating extensive dye degradation. This pronounced reduction in SERS signal demonstrates that the BiVO_4_@AuNSt@MIP micromotors are not only photocatalytically active but also capable of in situ and real-time monitoring of pollutant degradation.

These results show that the combined plasmonic properties of AuNSt and the molecular recognition capabilities of the MIP layer enabled highly sensitive detection of R6G at concentrations as low as 0.5 ppm (Fig. 5E), demonstrating the synergistic effect of combining nanoplasmonics and molecular imprinting within a photocatalytic micromotor system. The superior sensitivity of BiVO_4_@AuNSt@MIP micromotors is further evidenced by the comparison between UV–Vis and SERS analyses: Although the R6G absorption signal is nearly undetectable by UV–Vis, a distinct SERS response remains observable.

To assess the reusability of BiVO_4_@AuNSt@MIP micromotors, we tested their photocatalytic activity over 3 consecutive cycles using the same particles (Fig. S11). The micromotors maintained high photocatalytic activity during the first 2 cycles (96.5% and 93.3%, respectively), but a noticeable decrease was observed in the third cycle, with the degradation efficiency dropping to 80.5%. This decline is likely associated with partial degradation or depletion of the MIP layer after repeated use, as previously reported in similar systems [11]. Figure S12 shows FESEM images of the micromotors before and after the photocatalytic reaction. The characteristic star-shaped BiVO_4_ morphology is fully preserved, indicating that the inorganic core remains structurally intact during the photocatalytic process. Prior to reaction, the micromotor surface appears relatively smooth, whereas after degradation, an increased surface roughness is observed, which is attributed to partial deterioration of the MIP polymer layer rather than changes in the BiVO_4_ structure. Moreover, the zeta potential shifted from −11.3 to −44.7 mV after the reaction, reflecting the loss of positively charged functional groups in the polymer layer and/or adsorption of charged degradation by-products. Importantly, such polymer degradation does not represent a permanent limitation, as similar micromotor systems have been shown to be readily refunctionalized, enabling regeneration of the polymeric coating and recovery of performance [11,78,79].

Overall, while acknowledging the need to improve their long-term operational stability, the potential of BiVO_4_@AuNSt@MIP micromotors for pollutant removal and real-time monitoring is clearly demonstrated, therefore becoming a suitable platform for environmental remediation. Future studies will explore the detailed physicochemical properties of the MIP layer, including thickness and cross-linking density. Moreover, in situ overgrowth of colloidally prepared gold seeds enabled us to grow AuNSt directly on the surface of the BiVO_4_ micromotors, making it possible to tune the AuNSt coverage from “low” to “high”. However, it may be possible to improve AuNSt synthetic control even further by developing a “fully in situ” growth approach [80], where each synthesis step is carried out directly on the micromotor surface, avoiding colloidal growth and seed attachment steps.

## Conclusion

We demonstrate multifunctional micromotors that combine selective recognition, photocatalytic degradation, and real-time SERS detection within a single light-driven platform. By decorating star-shaped BiVO_4_ micromotors with plasmonic AuNSt formed in situ through a seed-mediated growth strategy and subsequently functionalizing them with a molecularly imprinted polymer layer, we achieve enhanced visible-light-driven propulsion, highly selective pollutant targeting, and ultrasensitive SERS-based detection down to trace levels. This unique combination of photocatalysis, recognition, and detection not only boosts degradation efficiency for model pollutants such as R6G but also enables in situ monitoring of the degradation process with molecular precision. The modular nature of the imprinting strategy potentially allows easy reprogramming for different targets, making these micromotors a highly adaptable tool for tackling a broad range of environmental contaminants. Together, our results introduce both a scalable synthetic route and a multifunctional design concept for smart, light-responsive micromotors for environmental remediation, paving the way toward next-generation microrobotic systems capable of navigating and sensing complex chemical landscapes with precision.

## Materials and Methods

### Materials

Analytical grade reagents, HAuCl_4_·3H_2_O (≥99.9%), silver nitrate (AgNO_3_; ≥99.9%), l-ascorbic acid (99%), hydrochloric acid (HCl; ACS reagent, 37%), Triton X-100 (laboratory grade), CTAC (25 wt % in H_2_O), sodium borohydride (NaBH_4_; 99%), APTES (99%), and 200 proof ethanol (≥99.8%), were purchased from Merck. Bismuth(III) nitrate pentahydrate (≥99.9%) and sodium metavanadate (≥99.9%) were purchased from Sigma-Aldrich, ethylenediaminetetraacetic acid disodium salt dihydrate (Na_2_-EDTA; ≥99.9%) was from Apollo, and OPDA (98%) was from Thermo Fisher Scientific.

All glassware, lids, and magnetic stir bars used for synthesis were cleaned thoroughly with aqua regia and rinsed thoroughly with Milli-Q water before use.

### Methods

#### BiVO_4_ synthesis

Star-shaped BiVO_4_ micromotors were synthesized by a previously reported hydrothermal reaction with minor modifications [14]. Briefly, 0.55 g (1.5 mmol) of Na_2_-EDTA salt was dissolved in 25 ml of ethylene glycol at 100 °C while stirring. Once dissolved, 0.48 g (1 mmol) of Bi(NO_3_)_3_ was added to the solution and stirred until dissolved. Subsequently, 0.12 g (1 mmol) of NaVO_3_ was dissolved in 15 ml of water and added dropwise to the previous solution. The final solution was incubated for 7 d and then placed in a 50-ml Teflon container and subjected to hydrothermal treatment at 180 °C for 36 h. The product was then filtered and washed 3 times, each with water and ethanol, and then dried at 60 °C.

#### Growth of gold nanoparticles on BiVO_4_ micromotors

All solutions were prepared using Milli-Q water, unless otherwise stated. A 100 mM CTAC solution and a 100 mM Triton X-100 solution were stored at room temperature. Other solutions, such as 50 mM HAuCl_4_, 100 mM ascorbic acid, and 10 mM AgNO_3_, were stored at 4 °C. The ascorbic acid and AgNO_3_ solutions were prepared fresh weekly. Meanwhile, 10 mM NaBH_4_ solution was prepared immediately prior to use (water added to the solid NaBH_4_ seconds before addition to the seeding solution), and 5% (v/v) APTES in ethanol was freshly prepared daily.

##### APTES coating of BiVO_4_ particles

Approximately 10 mg of BiVO_4_ particles was suspended in 9.5 ml of ethanol, in a 15-ml plastic centrifuge tube. The capped tube was heated at 60 °C for 15 min (with the sonication off). Then, the sonication was turned on, and 500 μl of 100% APTES was added. The suspension was sonicated for an additional 5-min period.

Note that at all functionalization steps (APTES coating, seeding, and overgrowth), the microparticles must be monitored to ensure that they are not depositing or collecting at the bottom of the centrifuge tube. The purpose of the sonication is to ensure this; however, additional manual shaking or vortexing of the tube may be necessary if the microparticles do not remain suspended in solution, depending on the efficacy of the specific sonicator.

Excess APTES was removed by washing the particles via centrifugation 3 times: The particles were centrifuged at 3,500 rpm for 2 min (these conditions were also used for all washing steps described below), and then supernatant was replaced with pure ethanol and sonicated for 5 min. The APTES-coated particles were then transferred to Milli-Q water. After 30 min, the seed attachment and overgrowth steps were performed.

##### Preparation of gold seeds

The seed solution (small colloidal gold nanoparticles) was prepared by mixing 4.7 ml of 100 mM CTAC with 25 μl of 50 mM HAuCl_4_ in a 20-ml glass vial containing a magnetic stir bar. The stirring speed of the gold solution was increased to over 1,500 rpm, and 300 μl of the NaBH_4_ solution was immediately and rapidly injected. The color of the solution changed from faint yellow to brown within seconds, indicating the reduction of Au^3+^ to Au^0^ and the formation of seeds. Stirring was stopped after the color change. The seed solution, characterized by a yellow–brown appearance, was used within 1 h to prevent changes in their size [81].

##### Seeding BiVO_4_ micromotors

The APTES-coated BiVO_4_ micromotors were washed 3 times with Milli-Q water via centrifugation to remove ethanol. For the low-coverage samples, the seed solution was diluted 10× in Milli-Q water, whereas the gold seed solution was used as prepared (nondiluted) for the high-coverage samples. The APTES-functionalized BiVO_4_ particles were suspended in either the 10× diluted or nondiluted seed solutions at 1 mg of BiVO_4_ microparticles/1 ml of seed solution and sonicated at room temperature for 15 min to ensure uniform coating. The seed-coated BiVO_4_ particles were finally washed 3 times with Milli-Q water by centrifugation and resuspended in 1 ml of 100 mM Triton X-100 in a 15-ml plastic centrifuge tube.

Note that these washing steps are important, and the sample should be briefly sonicated during each resuspension in Triton X-100 between centrifugation steps to ensure the removal of any nonattached seeds. Otherwise, free AuNSt will grow in solution away from the BiVO_4_ microparticle surface.

##### In situ growth of gold nanoparticles on BiVO_4_ micromotors

The BiVO_4_-bound seeds were immediately overgrown: Similar to the APTES functionalization step, the ratio of growth solution to BiVO_4_ was 1 ml:1 mg. The details below refer to synthesis with 10 mg of BiVO_4_ particles.

The AuNSt growth solution was prepared by mixing 100 μl of 50 mM HAuCl_4_, 100 μl of 10 mM AgNO_3_, and 300 μl of 1 M HCl for every 10 ml of 100 mM Triton X-100 solution in a separate 20-ml glass vial (not containing the seed-functionalized microswimmers). This solution was then diluted 4× in 100 mM Triton X-100 or used as is (nondiluted) to prepare the low- and high-coverage samples, respectively, to a final volume of 9 ml. The growth process was initiated by adding either 80 or 320 μl (for the low- and high-coverage samples, respectively) of 100 mM ascorbic acid to the corresponding 4× diluted or nondiluted growth solutions under vigorous magnetic stirring (1,000 rpm). The solutions turned from faint yellow to colorless within seconds, after which they were immediately added to the 15-ml centrifuge tubes containing the 1-ml BiVO_4_ particle suspensions (in 100 mM Triton X-100, containing 10 mg of the seed-functionalized microparticles). The tube was then sonicated for 5 min at room temperature. A blue–black tint developed, indicating AuNSt formation. The particles were immediately washed 3 times with Milli-Q water to halt the synthesis, remove unreacted materials, and remove the Triton X-100.

#### Surface molecular imprinting of BiVO_4_@AuNSt micromotors

The BiVO_4_@AuNSt@MIP micromotors were fabricated through surface molecular imprinting, using OPDA as the monomer and R6G as the template molecule [11]. Specifically, 0.06 g of OPDA was dissolved in 10 ml of a 5-ppm R6G. Subsequently, 0.1 g of BiVO_4_@AuNSt was introduced into the solution, and the pH was adjusted to 2 with a 1 M HCl solution. The mixture was then exposed to irradiation using a 300-W OSRAM UV lamp for 1 h to trigger in situ polymerization. During this process, the color of the suspension changed from pale yellow to dark yellow, indicating polymer formation. The BiVO_4_@AuNSt@MIP micromotors were isolated by centrifugation and thoroughly washed with water to remove any residual reagents.

#### Photocatalytic degradation experiments

In a typical experiment, 5 mg of the micromotors was dispersed in 5 ml of a 5-ppm R6G solution, and 500 μl of 0.1% H_2_O_2_ was added as fuel. The sample was illuminated for 120 min with a 300-W Xenon light source with a 400-nm long-pass filter (Asahi Spectra), while UV–Vis or Raman spectra were recorded at regular intervals.

Degradation experiments were also performed in the presence of specific scavengers: AgNO_3_ (final concentration, 10 mM) as an e^−^ quencher, 2-propanol (final concentration, 10 mM) as an ·OH quencher, Na_2_-EDTA (final concentration, 0.5 mM) as a h^+^ quencher, and *p*-benzoquinone (final concentration, 0.5 mM) as a ^•^O_2_^−^ quencher. All the experiments were performed under 1 h of light irradiation.

#### Characterization of BiVO_4_-based micromotors

Surface morphology and chemical composition of BiVO_4_ micromotors were examined using FESEM equipped with a Ga ion beam (Scios 2, FEI) coupled with an EDS detector. A JEOL JSM 6490 LV SEM equipped with a top-view backscatter electron detector (scintillator–photomultiplier detector design) and an in-chamber side-view secondary electron detector (Everhart–Thornley design) was used to characterize the morphology of the prepared gold nanostructures on the microparticles (5-kV acceleration voltage). Optical properties and the respective bandgap calculations of micromotors were assessed using a UV–Vis spectrophotometer equipped with an integrating sphere (UV-2401PC, Shimadzu). The collected data were processed using the Kubelka–Munk function to obtain the absorption characteristics. To estimate the bandgap, a Tauc plot was constructed by plotting the transformed absorbance data against photon energy. The bandgap value was extracted by extrapolating the linear region of the plot to the energy axis, allowing for the identification of the optical transition characteristics of BiVO_4_. Raman spectroscopy for SERS measurements was performed with a Renishaw InVia Raman microscope equipped with a 785-nm laser, a 50× N PLAN L objective (50×/0.50, Leica), and a laser power of 7.12 mW at the sample (exposure time, 20 s; number of acquisitions, 3). Powder XRD data were collected on a Bruker AXS D8-Discover diffractometer. Zeta potential measurements of the powder samples suspended in ethanol were obtained using a Zetasizer Nano ZS (ZEN 3600, Malvern). TGA was performed in a TGA/SDTA851 (Mettler Toledo) thermogravimetric balance in a range of 40 to 550 °C using N_2_ atmosphere. The motion of the micromotors was analyzed from the recorded videos at 25 frames/s using a THUNDER Imager DMi8 inverted microscope (Leica), with a 63× objective, in time-lapse mode. The recording duration was set to 10 s, after which a tracking software was used to analyze and quantify their movement.

## Data Availability

The datasets supporting this article have been uploaded as part of the Supplementary Materials.
