# Supplementary material for: Nanoengineered Photoactive Micromotors for Targeted Pollutant Capture, Degradation, and SERS-Based Detection
Source: Research (Wash D C). 2026 Feb 9;9:1110. doi: 10.34133/research.1110 (PMC12883708; doi:10.34133/research.1110)
Supplement: Supplementary 1 — Figs. S1 to S12 Tables S1 and S2 Movies S1 to S4 [file research.1110.f1.zip › Supplementary Information.docx]

**Supplementary information**

Nanoengineered Photoactive Micromotors for Targeted Pollutant Capture, Degradation and SERS-based Detection

**Authors**

Viktoria D. Lovasz^1,2^ ^‡^, João M. Gonçalves^1^ ^‡^, Gail A. Vinnacombe-Willson^3,4^, Luis M. Liz-Marzán^3,4,5^*, Katherine Villa^1, 6^*

**Affiliations**

^1^Institute of Chemical Research of Catalonia (ICIQ-CERCA), The Barcelona Institute of Science and Technology (BIST), Tarragona, Spain

^2^Universitat Rovira i Virgili (URV), Campus Sescelades, Tarragona, Spain

^3^CIC biomaGUNE, Basque Research and Technology Alliance (BRTA), Donostia-San Sebastián, Spain

^4^CIBER de Bioingeniería, Biomateriales y Nanomedicina (CIBER-BBN), Donostia-San Sebastián, Spain

^5^Ikerbasque, Basque Foundation for Science, Bilbao, Spain

^6^ Institució Catalana de Recerca i Estudis Avançats (ICREA), Barcelona, Spain

‡ These authors contributed equally.

Address correspondence to: [llizmarzan@cicbiomagune.es](mailto:llizmarzan@cicbiomagune.es) ; [kvilla@iciq.es](mailto:kvilla@iciq.es)


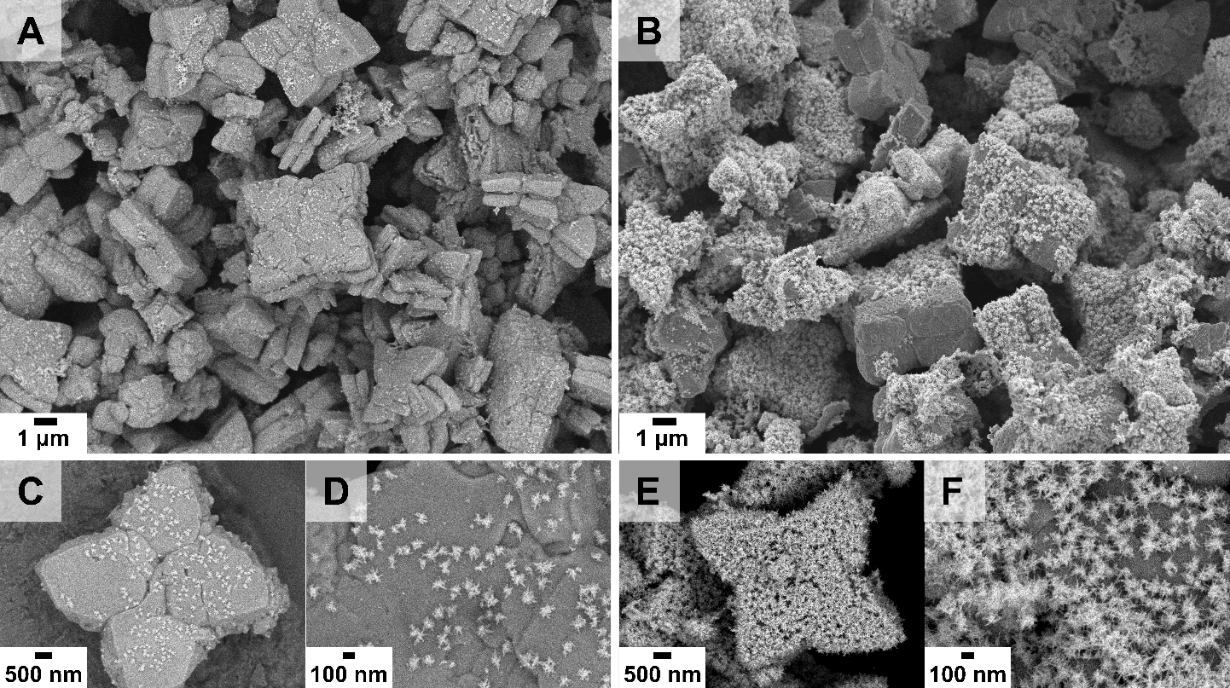


**Fig. S1.** SEM images of BiVO_4_ micromotors with different AuNSt coverage densities: (**A, C, D**) low coverage and (**B, E, F**) high coverage.


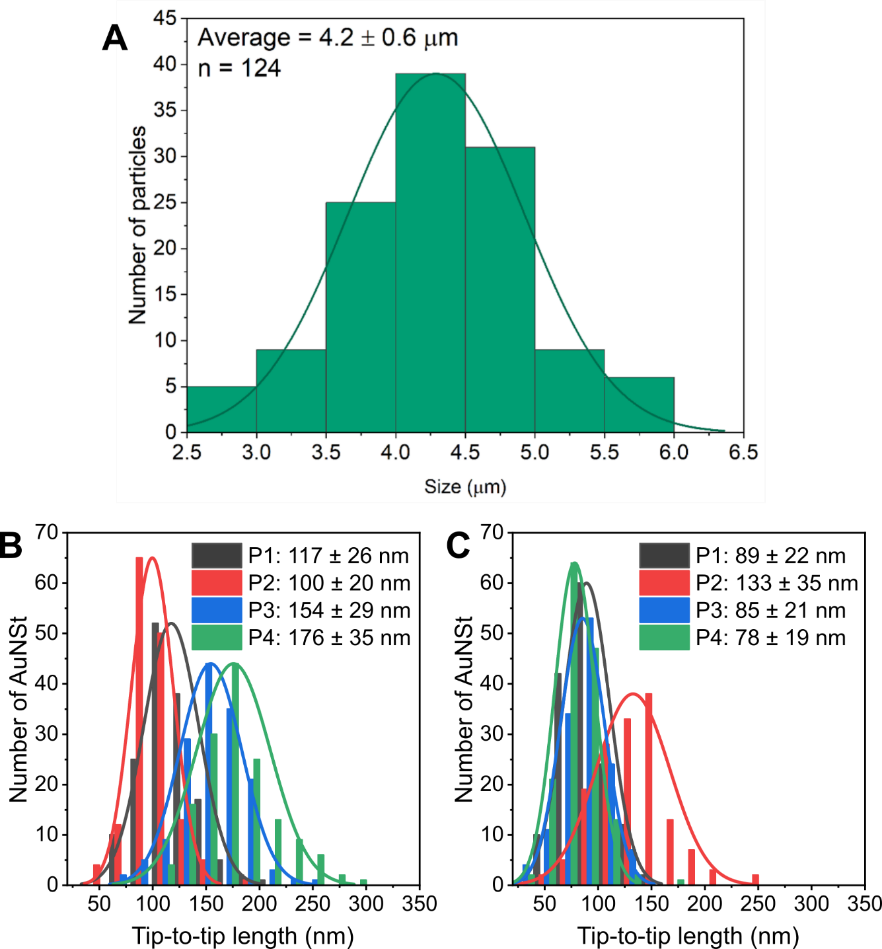


**Fig. S2.** (A) Size distribution of the BiVO_4_ micromotors based on measurements of 124 particles. (B) Tip-to-tip length distribution of AuNSt for high-coverage samples, measured across four different micromotors (*n* = 150 AuNSt per micromotor). The average size across the four samples is 140 ± 30 nm. (C) Tip-to-tip length distribution of AuNSt for low-coverage samples, measured across four different micromotors (n = 150 AuNSt per micromotor, except for P3 with *n =* 133). The average size across the four samples is 100 ± 30 nm.


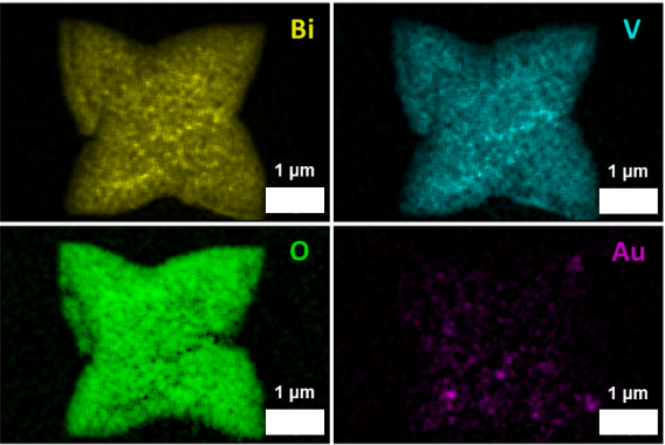


**Fig. S3.** EDS elemental mapping of BiVO_4_@AuNSt micromotors showing the distribution of Bi, V, O, and Au elements.


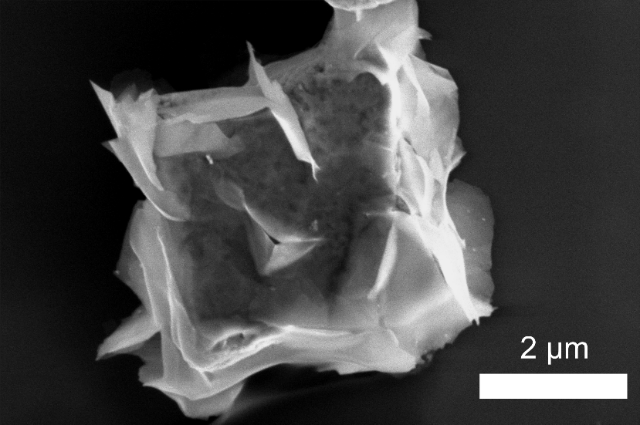


**Fig. S4.** FESEM image of BiVO_4_@AuNSt@MIP micromotors after surface modification with the molecularly imprinted polymer layer.


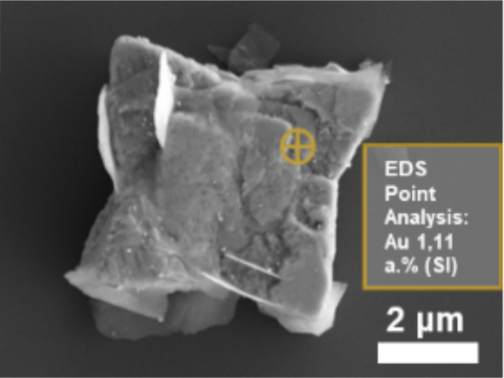


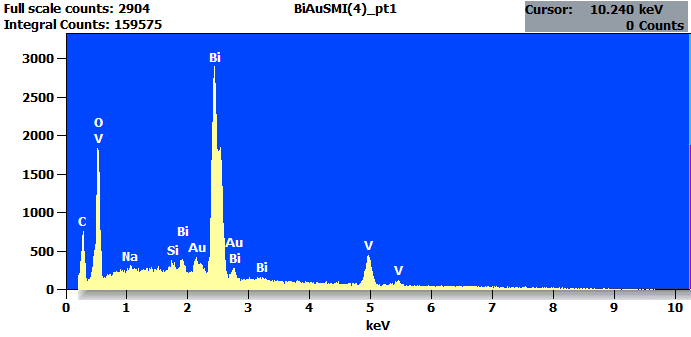


**Fig. S5.** Point EDS analysis of BiVO_4_@AuNSt@MIP micromotors. The FESEM image indicates the analysis site, while the corresponding EDS spectrum confirms the presence of Au with characteristic peaks, verifying that AuNSt remain on the micromotor surface after MIP coating.


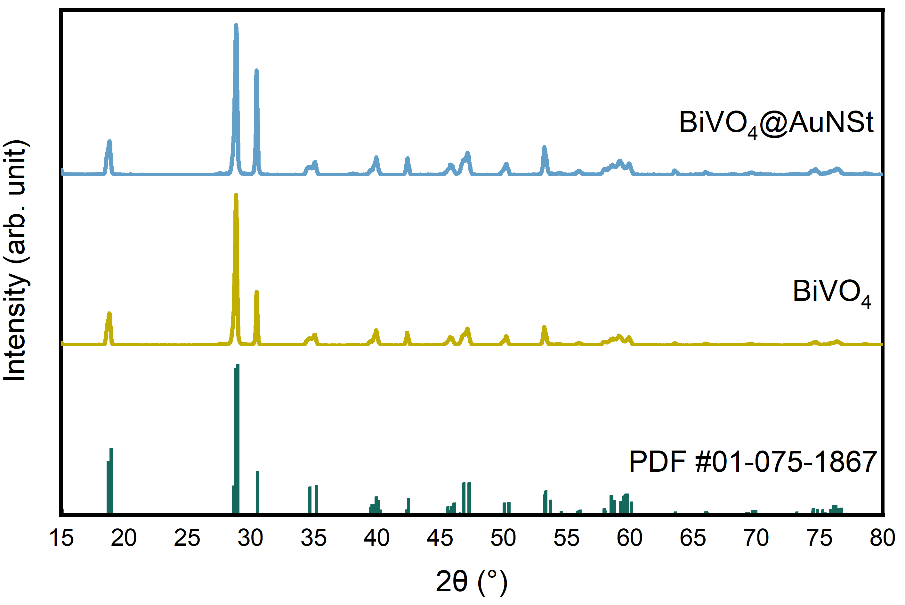


**Fig. S6.** XRD patterns of BiVO_4_@AuNSt and pristine BiVO_4_, showing the characteristic peaks of monoclinic BiVO_4_ phase (PDF: 01-075-1867).


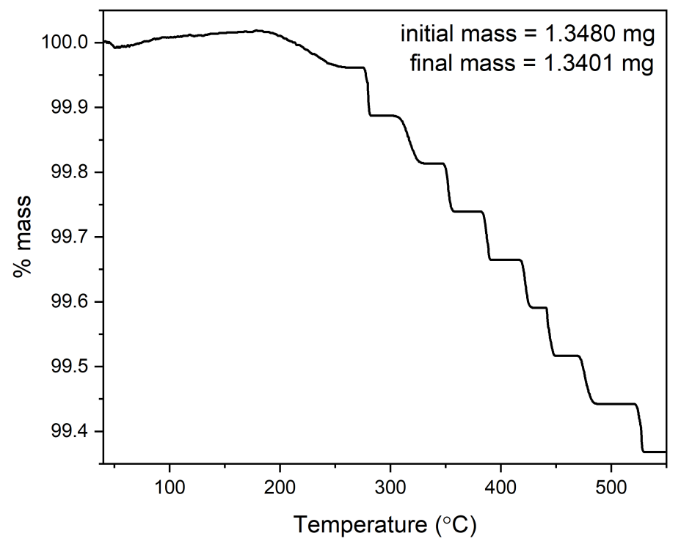


**Fig. S7.** Thermogravimetric analysis of BiVO_4_@AuNSt@MIP.


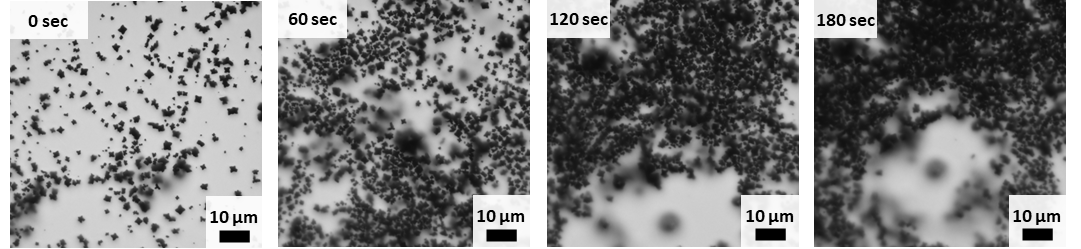


**Fig. S8.** Time-lapse images of BiVO_4_ micromotors recorded under 390 nm light irradiation, showing photo-induced clustering behavior.


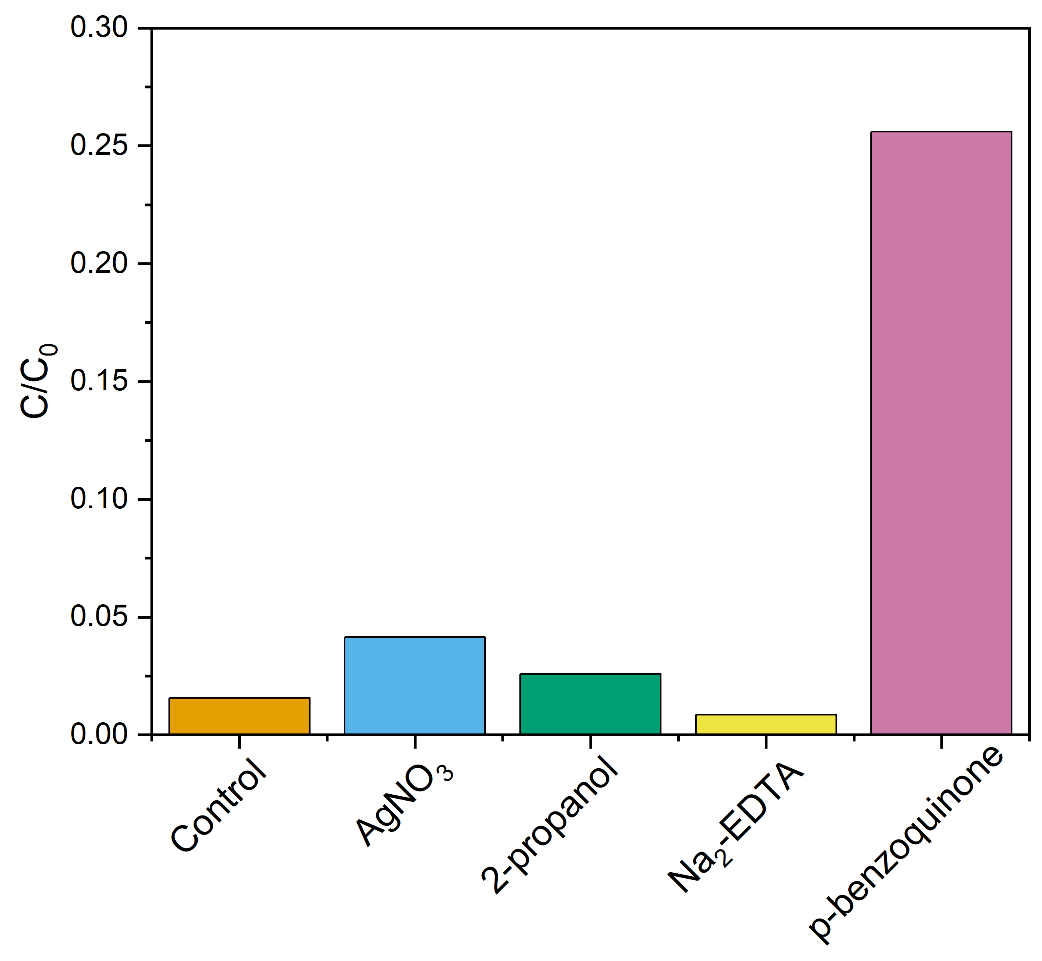


**Figure S9.** Photocatalytic degradation of R6G in the presence of radical scavengers to investigate the degradation mechanism. AgNO_3_ (e^−^ scavenger), 2-propanol (**^•^OH** scavenger)**, Na_2_-EDTA (h^+^** scavenger), **and p-benzoquinone (^•^O_2_^−^** scavenger).


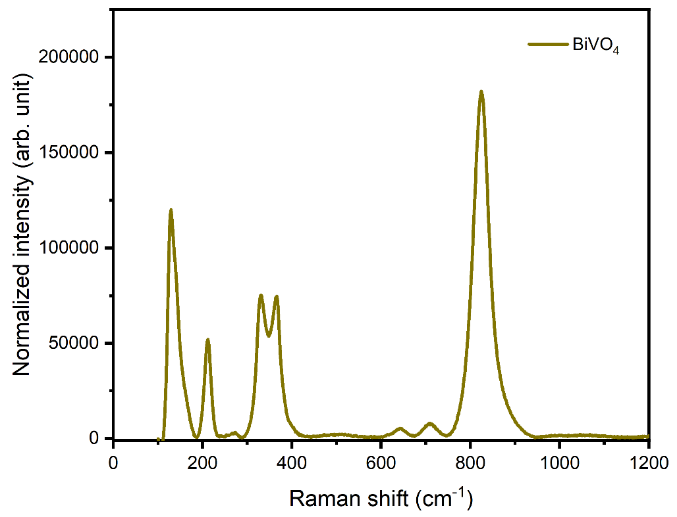


**Fig. S10.** Raman spectrum of BiVO_4_ micromotors in a 5 ppm Rhodamine 6G solution. The observed bands correspond exclusively to the characteristic vibrational modes of BiVO_4_, with no detectable contribution from R6G.


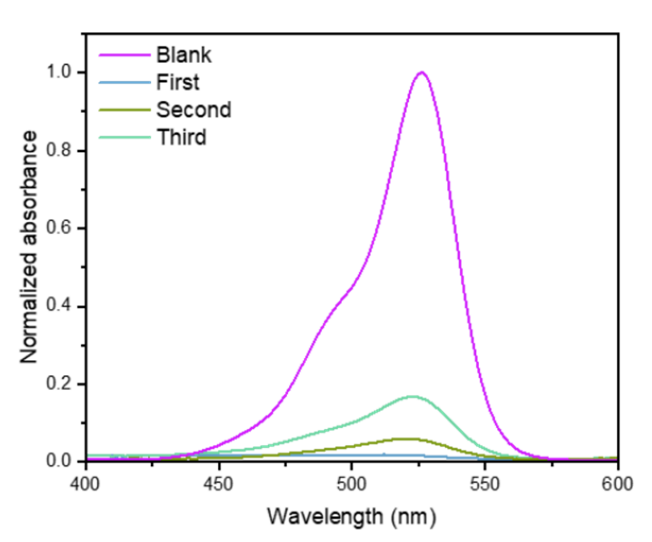


**Figure S11.** Photocatalytic degradation of R6G over sequential reaction cycles using BiVO_4_@AuNSt@MIP micromotors.


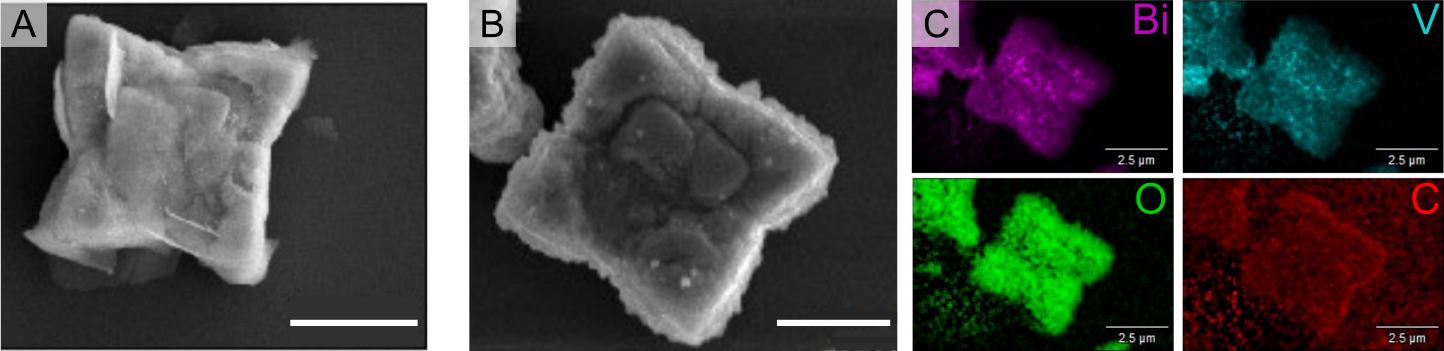


**Figure S12.** FESEM images of BiVO_4_@AuNSt@MIP. (A) Before any photocatalytic degradation cycle and (B) After one photocatalytic degradation cycle. (C) EDS mapping showcasing the elements found after one photocatalytic degradation cycle. Scale bars represent 2 μm, unless stated otherwise.

**Table S1**. Obtained velocities of BiVO_4_ micromotors with different AuNSts coverage densities

|  | 390 nm excitation | 475 nm excitation |
| --- | --- | --- |
| BiVO_4_ | 2.38 ± 0.33 μm/s | 1.77 ± 0.20 μm/s |
| BiVO_4_@AuNSt low coverage | 3.61 ± 0.44 μm/s | 2.70 ± 0.26 μm/s |
| BiVO_4_@AuNSt high coverage | 4.29 ± 0.37 μm/s | 4.74 ± 0.40 μm/s |

**Table S2**. Comparison between BiVO_4_@AuNSt@MIP micromotors with other systems found in the literature that also achieved decomposition of R6G

| Type of micromotor | Propulsion mechanism | Fuel conc. | Additive | | Excitation | | Velocity (μm/s) | | Performance | | Ref. |
| --- | --- | --- | --- | --- | --- | --- | --- | --- | --- | --- | --- |
| TiO_2_-Fe Janus spheres | Photocatalytic bubble production | 5% wt. H_2_O_2_ | | 1% wt. SDS | | UV (motion + degradation) | | 260 | | 95% in 12 min. | ^70^ |
| ZIF-8/ZnO/silver nanoparticles spheres | Chemical decomposition of H_2_O_2_ | 5% - 30% w/v H_2_O_2_ | | 0.01% - 2% w/v SDS | | UV (degradation only) | | 1109 | | 48.9% in 32 minutes | ^69^ |
| mSiO_2_@TiO_2_@PtNPs microtubes | Chemical decomposition of H_2_O_2_ | 2.5% wt. H_2_O_2_ | | 0.125% wt. SDS | | UV (degradation only) | | 970 | | 98% in 30 minutes | ^72^ |
| Co/Zn bimetallic Janus dodecahedrons | Light induced self-electrophoresis | 0.01 wt% H_2_O_2_ | | --- | | UV (motion + degradation) | | 5.2 | | 95% in 20 minutes | ^71^ |
| Pt/Co-Pi/BiOI/ZnO/rGO layeres microtubes | Bubble propulsion from chemical decomposition of H_2_O_2_ | 3% wt. H_2_O_2_ | | --- | | Visible light (435 nm laser – degradation only) | | 63.1 | | 94% in 60 minutes | ^75^ |
| MnO_2_ microspheres | Bubble propulsion from chemical decomposition of H_2_O_2_ | 5% wt. H_2_O_2_ | | 0.1% wt. SDS | | Decomposition of dyes through chemical consumption of fuel by micromotors | | 551 | | 29% in 1h | ^74^ |
| Pt/Fe microtubes | Bubble propulsion from chemical decomposition of H_2_O_2_ | 15% wt. H_2_O_2_ | | 0.5% wt. SDS | | Decomposition of dyes through chemical consumption of fuel by micromotors | | -- | | 88% in 6h | ^73^ |
| BiVO_4_@AuNSt@MIP | Light induced self-electrophoresis | 0.1% wt. H_2_O_2_ | | --- | | Visible light (475 nm LED – propulsion + degradation) | | 4.74 | | 96.5% in 90 min | This work |
